# Maternal Consumption of a Low-Isoflavone Soy Protein Isolate Diet Accelerates Chemically Induced Hepatic Carcinogenesis in Male Rat Offspring

**DOI:** 10.3390/nu12020571

**Published:** 2020-02-22

**Authors:** Jihye Choi, Sae Bom Won, Young Hye Kwon

**Affiliations:** 1 Department of Food and Nutrition, Seoul National University, Seoul 08826, Korea; nana7736@naver.com; 2 Department of Human Nutrition and Food Science, Chungwoon University, Hongseong, Chungnam 32244, Korea; newspring@chungwoon.ac.kr; 3 Research Institute of Human Ecology, Seoul National University, Seoul 08826, Korea

**Keywords:** apoptosis, hepatic carcinogenesis, maternal diet, rat offspring, soy protein isolate

## Abstract

It has been reported that maternal nutrition determines the offspring’s susceptibility to chronic diseases including cancer. Here, we investigated the effects of maternal diets differing in protein source on diethylnitrosamine (DEN)-induced hepatocarcinogenesis in adult rat offspring. Dams were fed a casein (CAS) diet or a low-isoflavone soy protein isolate (SPI) diet for two weeks before mating and throughout pregnancy and lactation. Offspring were weaned to and fed a chow diet throughout the study. From four weeks of age, hepatocellular carcinomas (HCC) were induced by intraperitoneal injection of DEN once a week for 14 weeks. The SPI/DEN group exhibited higher mortality rate, tumor multiplicity, and HCC incidence compared with the CAS/DEN group. Accordingly, altered cholesterol metabolism and increases in liver damage and angiogenesis were observed in the SPI/DEN group. The SPI/DEN group had a significant induction of the nuclear factor-κB-mediated anti-apoptotic pathway, as measured by increased phosphorylation of IκB kinase β, which may lead to the survival of precancerous hepatocytes. In conclusion, maternal consumption of a low-isoflavone soy protein isolate diet accelerated chemically induced hepatocarcinogenesis in male rat offspring in the present study, suggesting that maternal dietary protein source may be involved in DEN-induced hepatocarcinogenesis in adult offspring.

## 1. Introduction

The “fetal basis of adult disease” hypothesis purports that chronic disease in adulthood is determined by in utero nutrition and other environmental factors, which may affect growth, development, and lifespan in mammals [1]. Especially, the altered cell proliferation, differentiation and apoptosis during fetal and neonatal life can modify the development of offspring’s organs, leading to long-term changes to the offspring’s physiology and metabolism [2]. Previous studies have mostly investigated the fetal programming mechanisms that link maternal undernutrition with impaired fetal growth and later development of cardiovascular disease and diabetes [3]. Since the liver is one of the major organs involved in coordinating metabolism, changes in liver development may contribute to a progression of liver disease upon a second hit [4]. 

Soybeans and soybean foods, which contain about 3.5 mg isoflavones per g of soy protein, have been shown to alleviate various chronic diseases [5]. Asian populations consume 20−50 mg of total isoflavones per day, while Americans consume less than 3 mg per day [6]. Previous studies have reported effects of early life genistein or isoflavone exposure on tumorigenesis, especially in mammary gland [7]; however, effects of early life soy protein isolate (SPI) exposure on tumorigenesis in adult offspring are yet to be fully explored. Compared with offspring fed a casein diet from gestation day 4 to postnatal day 194, offspring fed an SPI diet, had a decreased colon tumor incidence. However, SPI consumption only in utero increased the percentage of animals bearing multiple colon tumors, and this result was not observed in offspring fed casein supplemented with genistein (2.5 g/kg) during the same period [8]. In contrast, female offspring exposed to an SPI diet had increased mammary tumor latency and decreased multiplicity compared with those exposed to a casein diet in utero. In utero exposure to supplemented genistein (250 mg/kg) did not change tumor parameters [9]. These previous studies suggested that limited exposure to SPI diet during gestation regulates chemically induced tumorigenesis later in life, although conflicting observations in different types of tumors were reported. No studies have been conducted to investigate the association between maternal diet and liver tumor incidence. 

Hepatocellular carcinoma (HCC) is one of the most common malignancies worldwide [10] and represents a classic example of inflammation-related cancer [11]. The development of HCC is a multi-step process, usually progressing from regenerative nodules in cirrhosis. As cirrhotic liver cells accumulate chromosomal aberrations and genetic and epigenetic mutations, regenerative and dysplastic nodules advance to HCC [12]. The rat model of diethylnitrosamine (DEN) is one of the most accepted models for investigating hepatocarcinogenesis [13,14]. DEN is metabolized by cytochrome isoform 2E1 in the liver and generates reactive oxygen species [15]. Moreover, DEN forms alkyl DNA adducts and induces chromosomal aberrations, micronuclei, and sister chromatic exchange in the rat liver [16]. 

In comparison to a casein diet, maternal consumption of a low-isoflavone SPI diet altered hepatic transcriptome, especially genes involved in drug metabolism [17] and lipid metabolism [18], in our previous studies with 21-day-old rat offspring. These offspring also showed higher cell proliferation and mammalian target of rapamycin pathway activation and lower apoptosis compared with offspring of dams fed either a casein diet or a casein diet supplemented with genistein, which may contribute to a higher relative liver weight [17]. Therefore, we investigated the effect of protein source in maternal diet on the development of chemically induced HCC in rat offspring.

## 2. Materials and Methods 

### 2.1. Animals and Diets

**Figure 1:**
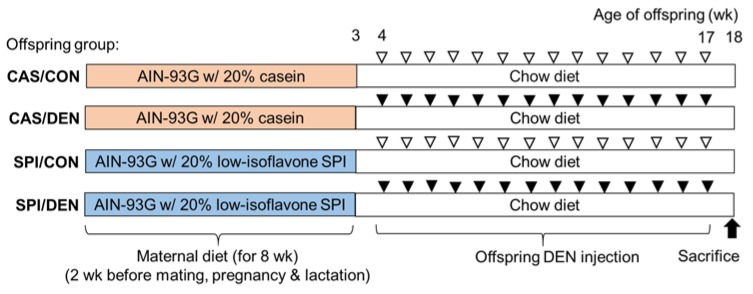
Experimental design. Dams were fed a casein diet (CAS) or a low-isoflavone soy protein diet (SPI) for two weeks before mating and throughout pregnancy and lactation. At three weeks of age, male offspring were separated from dams and acclimated to the regular chow diet. Four-week-old offspring from each set of dams received intraperitoneal injections of saline (▽) or diethylnitrosamine (DEN) at 50 mg/kg (▼) once a week for 14 weeks. At 18 weeks of age, male offspring were sacrificed.

Seven-week-old virgin female Sprague Dawley rats were purchased from Japan SLC, Inc. (Shizuoka, Japan). The experimental design is shown in Figure 1. After one week of acclimation, rats were randomly assigned to either a control casein diet (CAS diet; 20% casein) or a low-isoflavone SPI diet (SPI diet; 20% SPI). Experimental diets were isocaloric and were made according to the published AIN-93G formula [19], except that soybean oil was replaced with corn oil. SPI (PRO-FAM^®^ 974; Archer Daniels Midland Company, Chicago, IL, USA) used in the present study contained 7.47 mg genistein, 29.42 mg genistin, 2.73 mg daidzein, and 12.81 mg daidzin/kg diet, as previously reported [18]. Experimental diets were provided for two weeks before mating and throughout pregnancy and lactation. Diets and water were provided ad libitum. Mating was confirmed by detection of a vaginal plug and this day was denoted as day 0 of gestation and dams were then housed individually. The litter size and pup body weight were recorded at birth, and litters were adjusted to two females and six males to normalize the growth. 

After three weeks of lactation, offspring were acclimated to the regular chow diet. Four-week-old male offspring from each set of dams were divided into control (CON) and DEN-treated groups (CAS/CON; *n =* 6, CAS/DEN; *n* = 10, SPI/CON; *n* = 6, and SPI/DEN; *n* = 10). Offspring in DEN groups received intraperitoneal injections of DEN (Sigma, St. Louis, MO, USA) at 50 mg/kg body weight once a week for 14 weeks. A previous study showed that administration of DEN between 12 and 16 weeks induces the transition from a preneoplastic to a neoplastic state in rats [20]. At the same time, the CAS/CON and SPI/CON groups were injected with saline. Rats were maintained in a temperature- (22 ± 3 °C) and humidity- (50% ± 10%) controlled room, with a 12 h-dark-light cycle. The experimental protocol used in the present study was approved by the Institutional Animal Care and Use committee of Seoul National University (SNU-100706-1).

### 2.2. Tissue Sampling and Macroscopic Observations

Eight days after the 14th DEN-injection, male offspring were anaesthetized by an intraperitoneal injection of Zoletil 50® (Virbac, Fort Worth, TX, USA). Blood was rapidly obtained by cardiac puncture and was centrifuged at 3000 rpm for 20 min at 4 °C and stored at −80 °C until analysis. The total number of liver nodules with a diameter of 3 mm or more, which are detectable upon examination of the liver surface, was counted by two independent investigators according to the following criteria: Surface nodules with dyschromatic and dysmorphic patterns comprised the N1 (3 ≤ x < 5 mm in diameter), N2 (5 ≤ x < 10 mm in diameter), and N3 groups (x ≥ 10 mm in diameter), as previously reported [21]. The maximum diameter of each nodule was measured by using electronic calipers. After macroscopic evaluation, liver tissues were frozen immediately in liquid nitrogen and stored at –80 °C, or they were fixed in 10% buffered formalin. 

### 2.3. Liver Histology

Formalin-fixed liver tissue was processed into 4-μm-thick paraffin sections, which were stained with hematoxylin and eosin (H&E) and Masson’s trichrome for histopathology. All stained sections were evaluated by an experienced pathologist and were scored for inflammation, necrosis, steatosis, and fibrosis, as described in Supplementary Materials Table S1.

### 2.4. Serum Biochemical Analyses

Serum total bilirubin, total protein, and γ-glutamyl transferase (GGT) levels were measured with an Autodry chemistry analyzer (SPOTCHEM SP4410; Arkray, Inc., Kyoto, Japan). Serum total cholesterol, high-density lipoprotein (HDL) cholesterol, and albumin levels were determined using commercial kits (Asan Pharmaceutical Co., Seoul, Korea). The activities of serum alanine aminotransferase (ALT) and aspartate aminotransferase (AST) were measured by commercial enzyme kits (Asan Pharmaceutical Co., Seoul, Korea). Hepatic cholesterol levels were analyzed according to the method of Folch et al. [22]. Briefly, hepatic tissue was homogenized in phosphate buffered saline, incubated in methanol-chloroform (1:2, v/v), centrifuged at 1000× *g* for 15 min at 4 °C, and determined by the commercial kit used for serum.

### 2.5. Tissue Extract Preparation and Immunoblotting

Liver samples were homogenized in ice-cold lysis buffer, and the protein concentrations of lysates were determined using the Bio-Rad Protein Assay Reagent (Hercules, CA, USA). Equal amounts of protein were loaded into the lanes of an SDS-PAGE gel, separated, and blotted onto a PVDF membrane (Millipore, Burlington, MA, USA). After being blocked with 5% nonfat milk or bovine serum albumin (Sigma), the membrane was probed with a specific primary antibody for ATP binding cassette subfamily A member 1 (ABCA1; Abcam, Cambridge, U.K.), β-catenin (Santa Cruz Biotechnology, Inc., Dallas, TX, USA), cleaved caspase-3 (Cell Signaling Technology, Danvers, MA, USA), glyceraldehyde 3-phosphate dehydrogenase (GAPDH; Santa Cruz Biotechnology, Inc.), heat shock cognate protein 70 (HSC70; Santa Cruz Biotechnology, Inc.), lecithin cholesterol acyltransferase (LCAT; Abcam), phospho-IκB kinase α/β (p-IKKα/β; Cell Signaling Technology), liver type-fatty acid binding protein (LFABP; courtesy of Dr. Judith Storch, Rutgers, The State University of New Jersey, USA), or proliferating cell nuclear antigen (PCNA; Santa Cruz Biotechnology, Inc.). The membrane was then incubated with a specific horseradish peroxidase-linked secondary antibody (Sigma) for chemiluminescent detection. The band intensities were quantified with Quantity One software (Bio-Rad). 

### 2.6. Quantitative Real-Time PCR (qRT-PCR) Analyses

Total RNA from male offspring’s liver was isolated with Trizol (Invitrogen, Carlsbad, CA, USA) and the PureLink™ RNA Mini Kit (Invitrogen). To remove genomic DNA, total RNA was treated with PureLink™ DNase (Invitrogen) according to the manufacturer’s instruction. cDNA was synthesized from 2 μg of total RNA with the Superscript^®^II Reverse Transcriptase (Invitrogen). All amplification reactions were performed using a StepOne™ Real-Time PCR System (Applied Biosystems, Foster City, CA, USA) according to the manufacturer’s protocol. The following commercially available TaqMan® Assay primers and probes were purchased from Applied Biosystems: Rat c-myc (*Myc*, Rn00561507_m1), heme oxygenase-1 (*Hmox1*, Rn01536933_m1), interleukin 6 (*Il6*, Rn00561420_m1), matrix metalloproteinase 9 (*Mmp9,* Rn00579162_m1), and glyceraldehyde 3-phosphate dehydrogenase (*Gapdh*, Rn99999916_s1). Relative gene expression levels were analyzed using the 2^−ΔΔCt^ assay. 

### 2.7. Statistical Analyses

The data were analyzed using IBM SPSS statistics software (version 19, SPSS Inc., Chicago, IL, USA). The differences were evaluated with one-way ANOVA followed by Duncan’s multiple range test. For a comparison between two groups, either Student’s t-test or Mann-Whitney U test (a nonparametric test) was used. Data were expressed as mean ± SEM and differences were considered statistically significant at *p* < 0.05.

## 3. Results

### 3.1. Effects of Maternal Diet on Mortality Rate and Body Weight in DEN-Treated Rat Offspring

**Figure 2:**
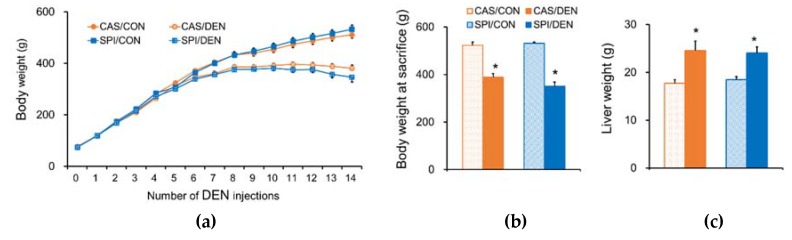
Effects of maternal diet on body weights in DEN-treated male rat offspring. (**a**) Body weight changes during DEN injection period. From four weeks of age, male offspring started to receive weekly intraperitoneal injection of DEN at 50 mg/kg. Body weights were measured weekly on the day of DEN or saline injection. (**b**) Body weight and (**c**) liver tissue weight at sacrifice. Results are expressed as means ± SEM (*n* = 6−10/group). ^*^Significantly different from the maternal diet-matched control (CON) group at *p* < 0.05 (one-way ANOVA with Duncan’s multiple comparison test).

Among ten offspring in each DEN-treated group, one offspring from the CAS/DEN group (after the 14th injection; 90% survival rate) and three offspring from the SPI/DEN group (one after the 13th injection and two after the 14th injection; 70% survival rate) died during the experimental period. The offspring’s body weight changes during the experimental period are shown in Figure 2a. The body weight was significantly reduced in both CAS/DEN and SPI/DEN groups compared with the corresponding control groups after the 6th injection. 

The final body weight in DEN-treated offspring was significantly lower compared with the maternal diet-matched CON group by 26% and 34% in the CAS and SPI groups, respectively (Figure 2b). Due to the appearance of liver nodules after DEN treatment, liver weights were significantly increased (Figure 2c).

### 3.2. Effects of Maternal Diet on HCC Incidence and Nodule Development in DEN-Treated Rat Offspring

**Figure 3:**
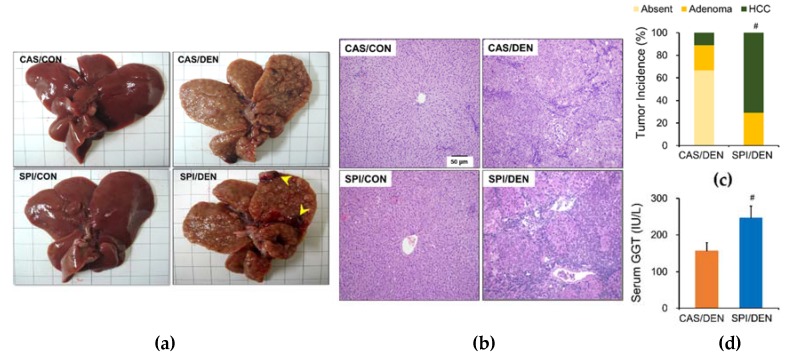
Effects of maternal diet on hepatocellular carcinoma (HCC) incidence and nodule development in DEN-treated male rat offspring. (**a**) Representative livers from control and DEN-treated rats. Regenerating nodules, early cancerous nodules, and malignant nodules with a diameter ≥ 3 mm (arrowhead) were detected on the surface of the liver. (**b**) Representative hematoxylin and eosin (H&E) stained liver sections from each group (magnification ×100). Photographs show the absence of HCC in CAS/CON, SPI/CON, and CAS/DEN groups and the presence of HCC in SPI/DEN group. (**c**) Liver cancer incidence based on histological examination. Incidence is expressed as percentage of the number of rats with designated tumor grade per total number of surviving rats. ^#^Significantly different from the CAS/DEN group at *p* < 0.05 (by Mann–Whitney U test). (**d**) Serum γ-glutamyl transferase (GGT) activity. Results are expressed as means ± SEM (n = 7 or 9/group). ^#^Significantly different from the CAS/DEN group at *p* < 0.05 (by Student’s *t*-test).

By the end of the experiment, gross examination showed that all DEN-injected rats had multiple hepatic nodules (Figure 3a). We observed pale yellow color, swelling, and preneoplastic and neoplastic lesions of various sizes, including small foci and gross nodules. Tumor multiplicity was higher in the SPI/DEN group compared with the CAS/DEN group (Table 1). Figure 3b shows the representative H&E stained liver sections of each group. Histological examination of H&E stained liver sections revealed a higher incidence of adenomas and HCCs in the SPI/DEN group compared with the CAS/DEN group (Figure 3c and Supplementary Materials Figure S1). Sections from the SPI/DEN group showed a loss of tissue architecture. In these sections, there were tumor cells that were smaller than normal cells and that contained granular cytoplasm and large hyperchromatic nuclei. We also observed significantly increased serum GGT levels in the SPI/DEN group (Figure 3d), confirming a higher incidence of HCC in the SPI/DEN group. As a sensitive biomarker of chemically induced HCC in rats, increased GGT is highly correlated with malignant transformation [20]. GGT was not detected in the control group.

Based on histological examination of H&E staining, there were no differences between the DEN-treated groups in the extent of liver necrosis, inflammation, and steatosis (Supplementary Materials Table S1). We also did not observe a significant difference in fibrosis in DEN-treated offspring, as determined by Masson’s trichrome staining (data not shown). Consistently, collagen type 1 mRNA levels were not significantly different between the DEN-treated groups (data not shown).

### 3.3. Effects of Maternal Diet on Liver Damage of DEN-Treated Rat Offspring

Serum biochemical parameters involved in liver function were shown in Table 2. There were no significant differences in liver damage parameters including AST, ALT, and total bilirubin between the CAS/DEN and SPI/DEN groups. However, DEN treatment significantly reduced serum total protein and albumin levels in the SPI group only, suggesting that the function of mature hepatocytes may be more severely damaged by DEN treatment in the SPI group compared with the CAS group.

### 3.4. Effects of Maternal Diet on Cholesterol Metabolism in DEN-Treated Rat Offspring

**Figure 4:**
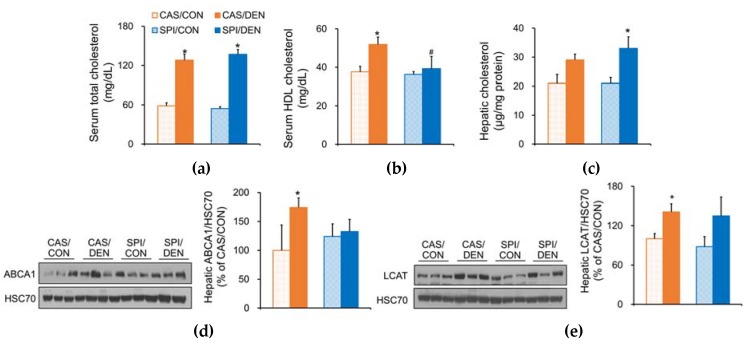
Effects of maternal diet on cholesterol metabolism in DEN-treated male rat offspring. Serum (**a**) total cholesterol and (**b**) high-density lipoprotein-cholesterol, and (**c**) hepatic cholesterol levels (*n* = 6−9/group). Hepatic protein levels of (**d**) ATP binding cassette subfamily A member 1 (ABCA1) and (**e**) lecithin cholesterol acyltransferase (LCAT) were determined by immunoblotting analysis, and were normalized to heat shock cognate protein 70 (HSC70) protein levels (*n* = 3/group). Results are expressed as means ± SEM. ^*^Significantly different from the maternal diet-matched CON group at *p* < 0.05. ^#^Significantly different from the CAS/DEN group at *p* < 0.05 (by one-way ANOVA with Duncan’s multiple comparison test).

Liver damage is associated with dysfunction of lipid metabolism, so we investigated serum and hepatic lipid profiles in DEN-treated offspring. Interestingly, we observed the altered cholesterol metabolism in the SPI group in response to DEN injection. Unlike serum total cholesterol levels, which were increased in response to DEN injection in both CAS and SPI groups (Figure 4a), serum HDL-cholesterol levels were significantly increased only in the CAS group (Figure 4b). In contrast, hepatic cholesterol levels were significantly increased only in the SPI group in response to DEN injection (Figure 4c). Accordingly, hepatic protein levels of LCAT, a key enzyme that is responsible for HDL production and the assembly, and ABCA1, a transporter that is responsible for cholesterol and phospholipid efflux, were significantly increased in offspring of dams fed a CAS diet in response to DEN injection. No significant changes in hepatic ABCA1 and LCAT protein levels were observed between the SPI/CON and SPI/DEN groups (Figure 4d,e). 

### 3.5. Effects of Maternal Diet on Cell Proliferation and Angiogenesis in the Liver of DEN-Treated Rat Offspring

**Figure 5:**
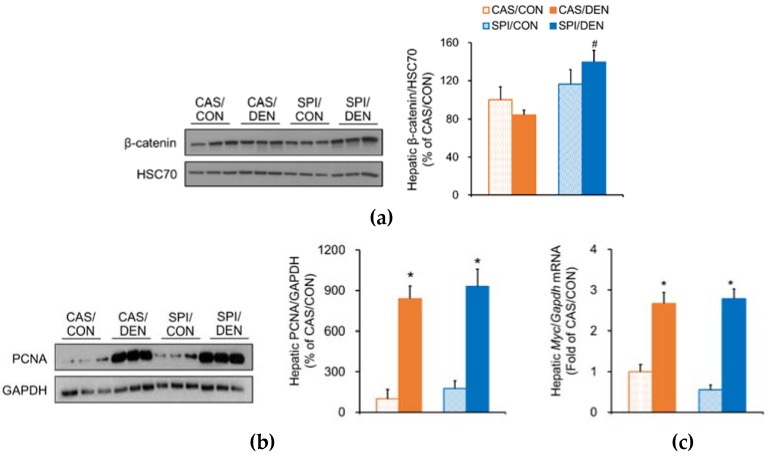

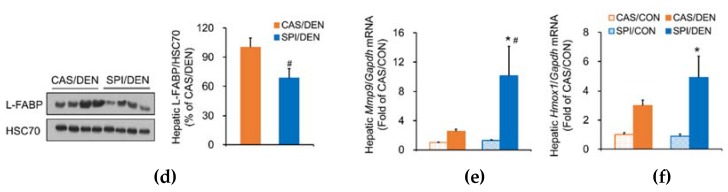
Effects of maternal diet on cell proliferation and angiogenesis in the liver of DEN-treated male rat offspring. Hepatic protein levels of (**a**) β-catenin, (**b**) proliferating cell nuclear antigen (PCNA), and (**d**) liver type-fatty acid binding protein (L-FABP) were determined by immunoblotting analysis, and were normalized to either heat shock cognate protein 70 (HSC70) or glyceraldehyde 3-phosphate dehydrogenase (GAPDH) protein levels (β-catenin and PCNA: n = 3/group, L-FABP: *n* = 7 or 9/group). Hepatic microRNA (mRNA) levels of (**c**) *Myc*, (**e**) *Mmp9,* and (**f**) *Hmox1* were determined by qRT-PCR, and were normalized to *Gapdh* mRNA levels (*n* = 4−9/group). Results are expressed as means ± SEM. ^*^Significantly different from the maternal diet-matched CON group at *p* < 0.05. ^#^Significantly different from the CAS/DEN group at *p* < 0.05 (by one-way ANOVA with Duncan’s multiple comparison test or by Student’s *t*-test for L-FABP).

In the SPI/DEN group, β-catenin protein levels were significantly higher compared with the CAS/DEN group (Figure 5a). The Wnt/β-catenin pathway is shown to be involved in the regulation of the development of HCC. Especially, in poorly differentiated tumors with increased cell proliferation and angiogenesis, an increased β-catenin accumulation in the nucleus and cytoplasm was observed [23]. Although we observed a significant increase in PCNA protein levels in the DEN-treated offspring, there was no significant difference between the CAS/DEN and SPI/DEN groups (Figure 5b). Similarly, mRNA levels of a cell proliferation marker gene, *Myc* was not significantly different between DEN-treated groups (Figure 5c). 

The SPI/DEN group exhibited significantly lower L-FABP protein levels than the CAS/DEN group, suggesting that the progressive loss of normal liver cells was accelerated in the SPI/DEN group (Figure 5d). *FABP1* coding for L-FABP is highly expressed in normal liver tissues compared with hepatocellular adenoma and HCC [24]. HCC is a hypervascular tumor that depends on angiogenesis for an adequate supply of oxygen and nutrients [25]. *Mmp9* mRNA levels were significantly increased by DEN only in the SPI group (Figure 5e). Similarly, expression of *Hmox1*, a gene involved in angiogenesis and anti-apoptosis, was also significantly increased in the SPI-DEN group (Figure 5f).

### 3.6. Effects of Maternal Diet on Activation of Anti-Apoptotic Signaling Pathway in DEN-Treated Rat Offspring

**Figure 6:**
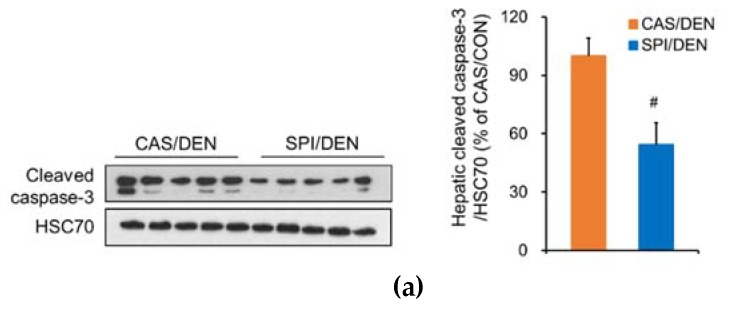

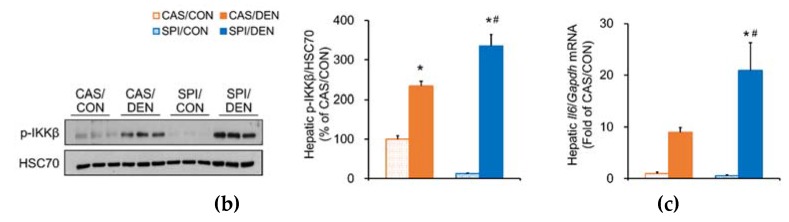
Effects of maternal diet on activation of anti-apoptotic signaling pathway in the liver of DEN-treated male rat offspring. Hepatic protein levels of (**a**) cleaved caspase-3 (*n* = 7 or 9/group) and (**b**) p-IKKβ (n = 3/group) were determined by immunoblotting analysis, and were normalized to heat shock cognate protein 70 (HSC70) protein levels. (**c**) Hepatic mRNA levels of ***Il6*** were determined by qRT-PCR, and were normalized to ***Gapdh*** mRNA levels (*n* = 4−9/group). Results are expressed as means ± SEM. ^*^Significantly different from the maternal diet-matched CON group at *p* < 0.05. ^#^Significantly different from the CAS/DEN group at *p* < 0.05 (by one-way ANOVA with Duncan’s multiple comparison test or by Student’s t-test for caspase-3).

The SPI/DEN group showed significantly reduced apoptosis compared with the CAS/DEN group, as determined by immunoblotting of cleaved caspase-3 (Figure 6a). Therefore, we investigated the regulation of signaling pathway involved in apoptosis. Nuclear factor κB (NF-κB) has been shown to promote inflammation-mediated development of liver cancer [26]. In the early stages of HCC development, the cytoprotective effect of NF-κB prevents cell death and inhibits compensatory cell proliferation by regulating gene expression of growth factors and cytokines. This activity may promote the survival of transformed hepatocytes into the late stages of tumorigenesis [27]. As determined by the increased levels of p-IKKβ (Ser177/181), the NF-κB pathway was significantly activated in the SPI-DEN group compared with the CAS-DEN group (Figure 6b). DEN treatment significantly increased *Il6* mRNA levels in the SPI group, but not in the CAS group (Figure 6c). IL-6 is one of the cytokines whose expression is controlled by NF-κB for cancer cell survival. In a previous study, DEN induced HCC development by promoting IL-6 production in preneoplastic liver tissues [11,28]. Similarly, anti-apoptotic activity of heme oxygenase (HO)-1 is known to require NF-κB-dependent gene transactivation in tumors [29]. *Hmox1* mRNA levels were significantly increased by DEN only in the SPI group, as shown in Figure 3e.

## 4. Discussion

In the present study, we investigated whether maternal dietary protein source may regulate chemically induced HCC development in offspring. Despite much research conducted on the role of maternal SPI diet in the development of chemically induced cancer in offspring, this is the first study, to our knowledge, that investigates the effect of SPI with low levels of isoflavone on liver carcinogenesis in offspring. Compared with the CAS group, there was an accelerated HCC development in the SPI group in response to DEN treatment based on higher mortality and HCC incidence. Furthermore, we observed significantly lower L-FABP protein levels in the SPI/DEN group compared with the CAS/DEN group. L-FABP expression is not detected in enzyme-altered foci positive for glutathione *S*-transferase P when carcinogenesis is initiated by DEN in rats [30]. 

Apoptosis was more inhibited in the SPI/DEN group than in the CAS/DEN group, which may be due to an inactivation of IKK and a subsequent activation of the NF-κB pathway. The activation of NF-κB signaling and induction of a target gene, *Il6*, were shown to promote the survival of cancer cells by protecting them against stress-induced cell death during the progression of inflammatory cancers, such as HCC and colitis-associated cancer [31]. In addition to anti-apoptotic effect, the NF-κB pathway in carcinogenesis is shown to increase expression of various invasion-related genes, including *Mmp2* and *Mmp9* [32]. Furthermore, the dynamic roles of activated NF-κB pathway in tumor growth and metastasis may be due to increases in expression of HO-1, which is shown to regulate inflammation, apoptosis and angiogenesis of cancer cells [33].

For several proliferation markers, no significant differences were observed between the CAS/DEN and SPI/DEN groups, which may be due to the increased liver regeneration in noncancerous animals. Hepatocyte death induced by chronic oxidative stress and inflammation triggers the restoration of the lost hepatic parenchyma. The robust regeneration of the liver involves all of its different cell types, and this reaction is likely evolved as a mechanism to preserve the liver’s essential ability to detoxify xenobiotics and endobiotics [11]. However, increased liver damage may result in a significantly higher incidence of HCC in the SPI/DEN group compared with the CAS/DEN group.

Consistent with our previous studies that reported that maternal consumption of low-isoflavone soy protein intake altered HDL metabolism in the 21-day-old rat offspring [18] and in adult offspring with alcoholic liver injury [34] compared to maternal casein consumption, we observed the altered HDL cholesterol metabolism in the present study. Since the liver is the major organ of lipoprotein synthesis and catabolism, serum lipid levels, particularly HDL-cholesterol, may be useful indicators in explaining the liver disease development, including HCC [35]. Accordingly, serum HDL-cholesterol levels were significantly decreased in HCC patients [36] and in DEN-injected rats [37] compared with the controls. 

Early consumption of soy has received considerable attention for their potential role in health and disease development [38]. Previously, we have reported that a maternal diet is associated with changes in gene expression and global DNA methylation patterns in livers from day 21 male offspring. Offspring of dams fed a low-isoflavone SPI diet showed increased cell proliferation and relative liver weight compared with the offspring of dams fed a casein diet, which was not observed in the offspring of dams fed a genistein diet (250 mg/kg diet). These results demonstrate that maternal consumption of a low-isoflavone SPI diet alters the hepatic gene expression profile and liver development of male offspring possibly by epigenetic processes [17]. Accordingly, distinct genes respond to maternal consumption of SPI or genistein in mammary epithelial cells of female offspring at day 50, which may contribute to the protective role of a maternal diet with SPI, but not a maternal diet with genistein alone, against mammary carcinogenesis in offspring [9,39].

## 5. Conclusions

Unlike soy isoflavone as a component of SPI or a dietary supplement ingredient, the effect of SPI with low levels of isoflavone on fetal programming has not been well understood. Here, we observed that the maternal consumption of a low-isoflavone SPI diet accelerated chemically induced hepatic carcinogenesis in male rat offspring, suggesting that a protein source in maternal diet may program DEN-induced hepatocarcinogenesis in adult offspring. Further analysis of aberrant gene expressions and their epigenomic regulation are necessary to determine the involved mechanism(s) by which the maternal diet may control apoptosis of precancerous cells.

## Figures and Tables

**Table 1:** Effects of maternal diet on body and organ weights of DEN-treated male rat offspring.

	Diet (Maternal/Offspring)			
	CAS/CON	CAS/DEN	SPI/CON	SPI/DEN
N1 (3 ≤ x < 5 mm)	0 ± 0	45.2 ± 7.0 ^*^	0 ± 0	63.7 ± 8.2 ^*#^
N2 (5 ≤ x <10 mm)	0 ± 0	6.1 ± 1.4 ^*^	0 ± 0	10.0 ± 2.5 ^*^
N3 (x ≥ 10 mm)	0 ± 0	0.4 ± 0.2	0 ± 0	0.4 ± 0.3
Multiplicity	0 ± 0	51.8 ± 8.0 ^*^	0 ± 0	74.1 ± 8.5 ^*#^

Data are means ± SEM (*n* = 6−9). ^*^Significantly different from the maternal diet-matched CON group at *p* < 0.05. ^#^Significantly different from the CAS/DEN group at *p* < 0.05 (by one-way ANOVA with Duncan’s multiple comparison test).

**Table 2:** Effects of maternal diet on serum biochemical parameters of DEN-treated male rat offspring.

	Diet (Maternal/Offspring)			
	CAS/CON	CAS/DEN	SPI/CON	SPI/DEN
AST (IU/L)	52.9 ± 2.0	92.3 ± 11.6 ^*^	54.2 ± 2.6	127.6 ± 27.6 ^*^
ALT (IU/L)	19.5 ± 1.3	49.7 ± 3.6 ^*^	20.4 ± 1.1	77.0 ± 29.9 ^*^
Total bilirubin (mg/dL)	0.45 ± 0.02	1.24 ± 0.22 ^*^	0.47 ± 0.02	1.73 ± 0.28 ^*^
Total protein (g/dL)	6.55 ± 0.18	6.36 ± 0.12	6.40 ± 0.10	5.73 ± 0.09 ^*#^
Albumin (g/dL)	4.83 ± 0.26	4.28 ± 0.09	4.87 ± 0.33	3.95 ± 0.18 ^*^

Data are means ± SEM (*n* = 6−9). ^*^Significantly different from the CAS/DEN group at *p* < 0.05. ^#^Significantly different from the maternal diet-matched CON group at *p* < 0.05 (by one-way ANOVA with Duncan’s multiple comparison test).

## Supplementary Materials

The following are available online at https://www.mdpi.com/2072-6643/12/2/571/s1. Supplementary Materials and Methods: Liver histology, Figure S1: Representative H&E stained liver sections of (A) adenoma and (B) hepatocellular carcinoma from DEN-treated male rat offspring (magnification ×200), Table S1: Effect of maternal diet on the liver pathology in DEN-treated male rat offspring.
