# Supplementary material for: Maternal Consumption of a Low-Isoflavone Soy Protein Isolate Diet Accelerates Chemically Induced Hepatic Carcinogenesis in Male Rat Offspring
Source: Nutrients. 2020 Feb 22;12(2):571. doi: 10.3390/nu12020571 (PMC7071430; doi:10.3390/nu12020571)
Supplement: Supplementary file 1 [file nutrients-12-00571-s001.pdf]

Supplementary Materials

Figure S1

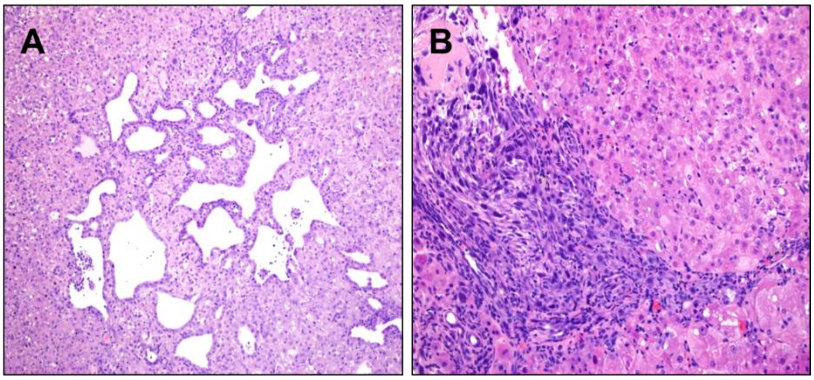

Representative H&E stained liver sections of (A) adenoma and (B) hepatocellular carcinoma from DEN-exposed offspring (magnification ×200).

Table S1. Effect of maternal diet on the liver pathology in DEN-treated male rat offspring

	Diet (Maternal/Offspring)			
	**CAS/CON**	**CAS/DEN**	**SPI/CON**	**SPI/DEN**
Necrosis[1]	0.0 ± 0.0	2.6 ± 0.2*	0.0 ± 0.0	3.0 ± 0.4*
Inflammation[2]	0.0 ± 0.0	2.7 ± 0.3*	0.0 ± 0.0	3.3 ± 0.3*
Steatosis[3]	0.0 ± 0.0	1.1 ± 0.1*	0.0 ± 0.0	1.0 ± 0.0*
Fibrosis[4]	0.0 ± 0.0	3.2 ± 0.2*	0.0 ± 0.0	3.6 ± 0.3*

[1] Focal (spotty) lytic necrosis was evaluated on H&E–stained sections and was given a score from 0 to 4 as follows: 0, absent; 1, 1 focus or less per 10× objective; 2, 2 to 4 foci per 10x objective; 3, 5 to 10 foci per 10× objective; 4, more than 10 foci per 10× objective.

[2] Portal inflammation was evaluated on H&E–stained sections and was given a score from 0 to 4 as follows: 0, no inflammation; 1, mild; 2, moderate; 3, moderate/marked; 4, marked.

[3] Steatosis was evaluated on H&E–stained sections and was given a score from 0 to 4 as follows: 0, absent; 1, less the 25%; 2, 25–50%; 3, 50–75%; 4, more than 75%.

[4] Fibrosis was evaluated on Masson's trichrome–stained sections and was given a score from 0 to 6 as follows: 0, absent; 1, fibrous expansion of some portal areas, with or without short fibrous septa; 2, fibrous expansion of most portal areas, with or without short fibrous septa; 3, fibrous expansion of most portal areas with occasional portal to portal (P-P) bridging; 4, fibrous expansion of portal areas with marked P-P bridging as well as portal-central (P-C) bridging; 5, marked bridging (P-P and/or P-C) with occasional nodules (incomplete cirrhosis); 6, cirrhosis, probable or definite.

Data are means ± SEM (n = 5–9). *Significantly different from the CAS/DEN group at $p < 0.05$. CAS; Casein; SPI; Low-isoflavone soy protein isolate; CON; Control; DEN; Diethylnitrosamine.
